# LncRNA CRNDE exacerbates neuropathic pain in chronic constriction injury-induced(CCI) rats through regulating miR-146a-5p/WNT5A pathway

**DOI:** 10.1080/21655979.2021.1972901

**Published:** 2021-10-06

**Authors:** Qiangze Zhang, Dongxia Zhu, Qiang Li

**Affiliations:** a Department of Pain, Ji’nan People’s Hospital Affiliated to Shandong First Medical University, Ji’nan, Shandong China; b Department of Traditional Chinese Medicine, Ji’nan People’s Hospital Affiliated to Shandong First Medical University, Ji’nan, Shandong China; c Department of Infectious Diseases Division, Laiwu People’s Hospital, Ji’nan, Shandong China

**Keywords:** Neuropathic pain, crnde, miR-146a-5p, wnt5a, lncRNA

## Abstract

Neuropathic pain (NP) originating from a dysfunction in the nervous system is often intractable and chronic. Many studies have implicated long noncoding RNAs (lncRNAs) in the physiological and pathological development of NP. The lncRNA colorectal neoplasia differentially expressed gene (CRNDE) has been shown to mediate NP progression. However, further investigations are needed to gain deeper understanding of the specific mechanisms governing CRNDE in NP etiopathology. In this study, we successfully used chronic constrictive injury (CCI)-induced rats to establish an NP model with intrathecal injection, and confirmed the upregulation of CRNDE in CCI-induced rats. Moreover, silencing of CRNDE relieved mechanical allodynia, thermal hyperalgesia, and neuroinflammation in the NP model. Bioinformatics analysis predicted that miR-146a-5p binds to CRNDE. Our findings validated that miR-146a-5p was a target of CRNDE and that the expression of miR-146a-5p was decreased in CCI rats. Furthermore, miR-151A-3p was found to exert a negative regulatory effect on WNT5A. In addition, knockdown of WNT5A alleviated the pain-related behavior and inflammatory response of NP *in vivo*. Finally, we demonstrated that CRNDE contributed to the progression of CCI-induced NP via competitive binding to miR-146a-5p to upregulate WNT5A. The present study offers novel insights that may be translated into improved therapies for NP.

## Introduction

Neuropathic pain (NP) is recognized as a kind of chronic pain which is persistent regular pain and leads to 11–19% of incidence in adult population [1,2]. NP is a significantly complex pain disorder arising from abnormalities in the somatosensory nervous system, reducing the life quality of patients and triggering their financial and mental burden [3–5]. It is estimated that approximately 6.9–10% of general public suffer from NP globally, and its morbidity trends to rise [6]. In view of the abstruse mechanism of NP development, it remains a challenge for physicians to improve the curative effect of NP. As a result, there is a pressing need to characterize therapeutic targets and illustrate the pathogenesis of NP.

Long non-coding RNAs (lncRNAs) are defined as a set of RNA molecules containing more than 200 nucleotides in length without the capacity to encode proteins [7,8]. A great number of reports have testified that lncRNAs are implicated in different biological activities of diverse disorders, including neurological disease [9,10]. Surging evidence has demonstrated that lncRNAs work as core modulators in the onset and evolution of NP [11]. For example, lncRNA GAS5 attenuates NP caused by chronic constriction injury (CCI) in vivo through targeting miR-452-5p/CELF2 pathway [12]. SNHG16 enhances JAG1 expression to exacerbate CCI-induced NP by competing for miR-124-3p and miR-141-3p [13]. Plentiful investigations have revealed that colorectal neoplasia differentially expressed gene (CRNDE) is identified as an oncogene in a variety of malignancies [14,15]. Notably, Zhang DW *et al*. uncovered that CRNDE played a stimulative role in the development of NP in CCI-induced rat models through regulation of miR‐136/IL6R axis [16]. Nevertheless, the molecular mechanism of CRNDE in neuropathologic progress has not been fully elucidated.

Wingless-related integration site 5A (WNT5A) located on chromosome 3p14-p21 belongs to the WNT family which is participated in activation of conventional Wnt signaling pathway [17]. It is well-known that Wnt signaling acts on a wide range of cell processes during the progression of neurological disease [18,19]. As a critical factor in Wnt pathway, WNT5A has been reported to provoke pain behaviors, enhance inflammatory response and sensibilize spinal nerves in rat models of NP [20,21]. However, the association between CRNDE and WNT5A in the occurrence and deterioration of NP remains indistinct.

We hypothesize that lncRNA CRNDE plays a key regulatory role in the progression of Neuropathic pain (NP), but the molecular mechanism of this effect is still unclear. To elucidate the mechanism of lncRNA CRNDE in Neuropathic pain (NP). we conducted a detailed study of lncRNA CRNDE in this experiment provides new insights for Neuropathic pain (NP), which can be transformed into improved NP therapy. Based on the foregoing descriptions, we undertook to lucubrate the involvement of CRNDE in NP progression. The goal of the current research was to explore the potential regulatory mechanism of CRNDE in the initiation and development of NP.

## Materials and methods

### Animal studies

Sprague-Dawley (8–2 weeks old, male) rats weighing 180–200 g were used in this study. All animals were maintained at an appropriate temperature (22 ± 4°C) under conditions of 12 h light-dark cycle and free access to food and water. All the animal protocols used in the current study complied with the standards for the use of laboratory animals. The experimental procedures were approved by the Animal Ethics Committee of Jinan People’s Hospital affiliated to the Shandong First Medical University.

### Establishment of an NP model

To construct an NP rat model, the animals underwent chronic constriction injury (CCI) surgery [5]. Briefly, following anesthesia using 10% chloral hydrate, the sciatic nerve was exposed via blunt dissection, and then the nerve near the sciatic trifurcation was ligated with 4–0 chromic gut sutures. Furthermore, the four ligation sites were 1 mm apart from each other. The surgical incision was sewn up with sutures, and the rats were monitored until they regained consciousness. Rats in the sham group were subjected to the same surgical procedure, except for nerve ligation. The spinal cord tissues were harvested from the rats following euthanasia.

### Intrathecal injection

Briefly, intrathecal injection was administered by inserting a sterile polyethylene-10 tubing into the rat lumbar region [13]. After fixation of the catheter, the surgical site was closed, and the externalized catheter was covered with a stainless-steel plug. The success of catheterization was evaluated with temporary hind paw paralysis following intrathecal injection of 20 µL lidocaine. To perform functional assays, lentiviruses carrying sh-CRNDE, WNT5A overexpressing vector, and the negative controls were synthesized by GenePharma (Shanghai, China) and were intrathecally injected into the rats using a microinjection syringe.

### Cell culture and transfection

Rat microglial cells were acquired from American Type Culture Collection (Rockville, USA) and maintained in DMEM (Gibco, CA, USA) containing 10% FBS (Gibco), 100 μg/mL streptomycin (Sigma, USA), and 100 U/mL penicillin (Sigma). All cells were cultured at 37°C in the presence of 5% CO_2_. For knockdown studies, sh-CRNDE and negative control scrambled shRNA were designed and supplied by GenePharma. Additionally, a pcDNA3.1 vector expressing CRNDE (Sangon Biotech, China) was used to upregulate CRNDE expression. Additionally, miR‐146a-5p mimic, miR‐146a-5p inhibitor, or negative control oligonucleotides were synthesized by GenePharma. Cell transfection was performed using Lipofectamine 2000 (Life Technologies, Carlsbad, CA, USA) according to the instructions [5].

### Nociceptive behavior test

The paw withdrawal threshold (PWT) was assessed to determine mechanical allodynia using Aesthesio Von Frey filaments (Bioseb, USA). The rats were adapted to a clear plastic box equipped with a metal mesh floor for approximately 1 h prior to the beginning of the experiments. Pressure was exerted on the sole of the rats via Von Frey filaments. Rapid flinch or paw withdrawal by the rats was identified as a positive response. The intensity of the stimulus was recorded when the rats exhibited a positive response [16].

To evaluate thermal hyperalgesia, heat and cold stimulation tests were performed. A radiant heat test instrument (Life Sciences, USA) was used to detect paw withdrawal latency (PWL). The cutoff time was set at 20 s to prevent tissue injury. PWL was determined as the time interval between the start of stimulation and retraction of the hind paw. Hypersensitivity in response to cold was assessed using the cold plate test. The rats were kept on a cold plate facility (Panlab, Spain), which was set at 4°C. The number of paw lifts was counted within 5 min following the beginning of the stimulation.

### Reverse transcription-quantitative PCR (RT-qPCR)

RNA was extracted using a TRIzol kit (Takara, Japan). Then, Prime Script^TM^ RT Master Mix (Takara) was used for cDNA synthesis. Gene expression were quantified by RT-qPCR in a CFX96 real-time PCR system (Applied Biosystems, USA) using the SYBR Premix Ex Taq II kit (Takara) according to the manufacturer’s instructions. GAPDH and U6 were used as normalization controls, and gene expression was calculated using the 2^−ΔΔCt^ method [13]. Primer sequences were shown: CRNDE, 5ʹ-CGCGCCCGCGCGGCGGAGGA-3ʹ- (forward), 5ʹ-AGTATGAATTGCAGACTTTGCA-3ʹ- (reverse); miR-146a-5p, 5ʹ-GGGGTGAGAACTGAATTCCAT-3ʹ- (forward), 5ʹ--CAGTGCGTGTCGTGGAGT-3ʹ- (reverse); WNT5A, 5ʹ-AGACGGGCATCAAAGAGT-3ʹ- (forward), 5ʹ-AAGCGGTAGCCATAGTC-3ʹ- (reverse); TNF-α, 5ʹ-CATGATCCGAGATGTGGAACTGGC-3ʹ- (forward), 5ʹ-CTGGCTCAGCCACTCCAGC-3ʹ- (reverse); IL-1β, 5ʹ-GGATAACGAGGCTTATGTGCACG-3ʹ- (forward), 5ʹ-GGACATGGAGAACACCACTTGTT-G-3ʹ- (reverse); IL-6, 5ʹ-GACTGATGTTGTTGACAGCCACTGC-3ʹ- (forward), 5ʹ-AGCCACTCCTTCTGTGACTCTAACT-3ʹ- (reverse); IL-10, 5ʹ--CGGGAAGACAATAACTGCACCC-3ʹ- (forward), 5ʹ-CGGTTAGCAGTATGTTGTCCAGC-3ʹ- (reverse); GAPDH, 5ʹ-CAAGGTCATCCATGACAACTTTG-3ʹ- (forward), 5ʹ-GTCCACCACCCTGTTGCTGTAG-3ʹ- (reverse); U6, 5ʹ-CTCGCTTCGGCAGCACATATACT-3ʹ- (forward), 5ʹ-ACGCTTCACGAATTTGCGTGTC-3ʹ- (reverse).

### RNA-binding protein immunoprecipitation (RIP)

RIP was performed using the Magna RIP RNA-Binding kit according to the manufacturer’s protocols [16]. RNA-binding proteins in the cell lysates were immunoprecipitated using an Ago2 antibody with IgG antibody as a negative control (Millipore, USA). The precipitated RNAs were quantified using the RT-qPCR assay.

### Luciferase reporter assay

Wild-type (Wt) or mutant (Mut) CRNDE or WNT5A were amplified and inserted into the pGL3 basic plasmid (Promega, USA). HEK-293 T cells were co-transfected with the constructed vectors and NC mimic or miR-146a-5p mimic using Lipofectamine 2000 (Life Technologies). Luciferase activity was tested using the Dual-Luciferase Reporter Assay System (Promega) [13].

### Enzyme-linked immunosorbent assay (ELISA)

The expression levels of tumor necrosis factor α (TNF-α), interleukin (IL)-1β, IL-6, and IL-10 in spinal cord tissues from different groups were measured using the corresponding ELISA kits (R&D Systems, USA) according to the instructions [16].

### Western blotting

Total protein was isolated from spinal cord tissues and microglial cells using RIPA lysis buffer (Thermo Fisher Scientific, Waltham, MA, USA) and quantified using the BCA method [21]. Equal amounts of proteins were separated using 10% sodium dodecyl sulfate polyacrylamide gel electrophoresis (SDS-PAGE), followed by transfer to polyvinylidene difluoride (PVDF) membranes and blocking in 5% skimmed milk. Next, the membranes were incubated with primary antibodies against WNT5A or GAPDH at 4°C overnight followed by washes and then probed with the corresponding secondary antibody at 37°C for 2 h. Protein bands were visualized using an enhanced chemiluminescence (ECL) kit (Beyotime, Shanghai) according to the manufacturer’s instructions. The protein expression of WNT5A was normalized to that of GAPDH.

### Statistical analysis

Data were analyzed and processed using SPSS 21.0 (IBM Corp., USA). All results are presented as the mean ± standard deviation (SD). Comparisons between two or more groups were analyzed using unpaired Student’s *t*-test or one-way ANOVA. Pearson’s correlation analysis was used to assess the relationship between gene expression. Statistical significance was set at *P* < 0.05.

## Results

In order to clarify the mechanism of lncRNA CRNDE in Neuropathic pain (NP), we conducted research on lncRNA CRNDE to provide new insights for Neuropathic pain (NP), which can be transformed into improved NP therapy. We hypothesize that lncRNA CRNDE plays a key regulatory role in the progression of Neuropathic pain (NP).Our study found that the expression of lncRNA CRNDE was up-regulated in rats induced by chronic constrictive injury (CCI), and confirmed that miR-146a-5p is the target of CRNDE. At the same time, the expression of miR-146a-5p in CCI rats was reduced and miR-151A-3p was found to have a negative regulatory effect on WNT5A.

## CRNDE expression was notably elevated in the NP rat model

**Figure 1.**
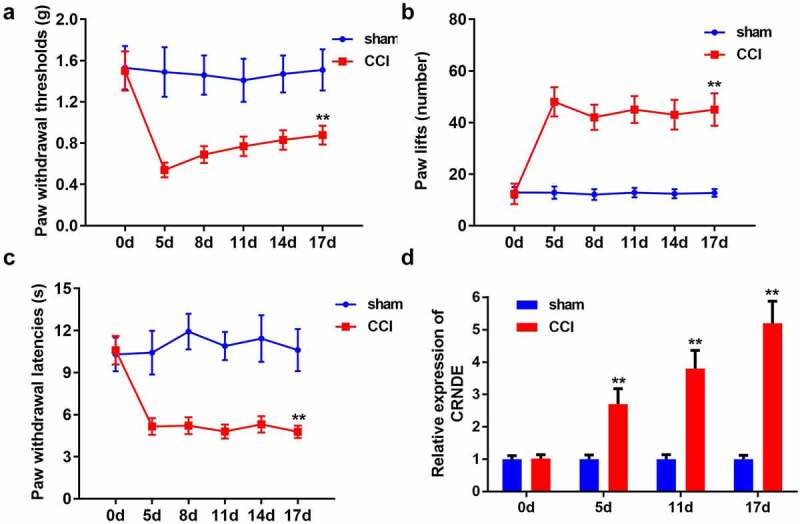
**CRNDE expression was notably elevated in the NP rat model**. (a) Behavioral nociceptive response to mechanical stimuli in CCI-induced rats was evaluated by PWT on different postoperative days. (b-c) Behavioral nociceptive response to thermal hypersensitivity in CCI-induced rats was estimated by paw lift number and PWL on different postoperative days. (d) RT-qPCR detection of CRNDE expression in spinal cord tissues from sham and CCI groups. **P < 0.01

To establish the NP model, rats underwent CCI surgery. Pain-related behaviors in the CCI-induced rats were estimated using the pain threshold test. As shown in Figure 1a, CCI led to a marked decrease in the PWT. The results revealed that the number of paw lifts was higher in the CCI-induced group than in the sham group (Figure 1b). Consistent with these results, the results of PWL assessment suggested that hypersensitivity in response to radiant heat was enhanced in the NP model (Figure 1c). In addition, the expression level of CRNDE was clearly upregulated in a time-dependent manner in the NP rat model established by CCI (Figure 1d). Collectively, we successfully established an NP rat model using the CCI method, and CRNDE was highly expressed in CCI-induced rats.

## Depletion of CRNDE contributed to the amelioration of NP in CCI rats

**Figure 2.**
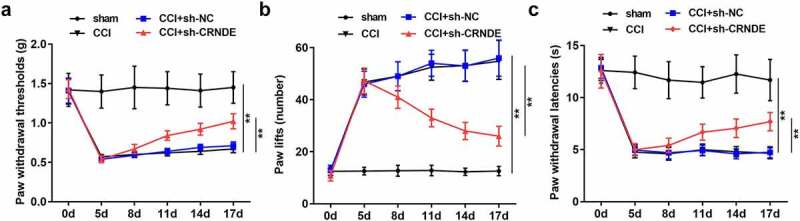
**Depletion of CRNDE contributed to the amelioration of NP in CCI rats**. (a) The effects of CRNDE knockdown on the mechanical allodynia in CCI rats were determined by PWT. (b-c) The effects of sh-CRNDE on the thermal hypersensitivity in CCI rats were assessed by the number of paw lift and PWL. **P < 0.01

We then sought to determine the effect of CRNDE on CCI-induced NP. For loss-of-function assays, the rats were intrathecally injected with lentivirus carrying sh-CRNDE. Knockdown of CRNDE resulted in the recovery of PWT induced by CCI surgery (Figure 2a). As expected, the number of paw lifts was reduced in the NP rat model owing to the inhibition of CRNDE (Figure 2b). Consistently, when compared to the rats in the CCI + sh-NC group, a marked increase in PWL was observed in the CCI + sh-CRNDE group (Figure 2c). Based on these results, we concluded that CRNDE downregulation relieved the NP-like behavior in CCI rats

## Silencing of CRNDE attenuated the inflammatory response *in vivo*

**Figure 3.**
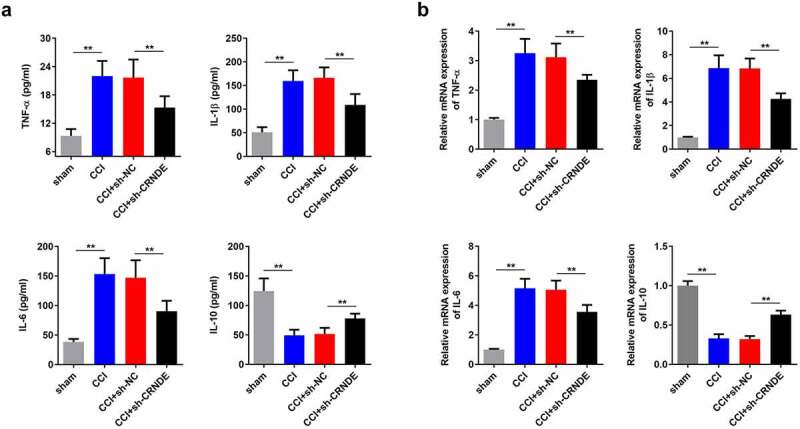
**Silencing of CRNDE attenuated inflammatory response *in vivo.*** (a) ELISA was performed to measure the concentrations of TNF-α, IL-1β, IL-6 and IL-10 in spinal cord tissues of rats from control and sh-CRNDE groups. (b) RT-qPCR analysis of TNF-α, IL-1β, IL-6 and IL-10 mRNA expression levels in control and sh-CRNDE groups. **P < 0.01

In view of the above findings, we further investigated the role of CRNDE in neuroinflammation in rats following CCI surgery by detecting the expression of inflammatory cytokines. ELISA results showed that CCI caused an increase in the levels of pro-inflammatory cytokines TNF-α, IL-1β, and IL-6, as well as a decrease in the expression of the anti-inflammatory cytokine IL-10. Suppression of CRNDE restored the expression of these inflammation-associated cytokines in the CCI rats (Figure 3a). Likewise, RT-qPCR analysis showed that knockdown of CRNDE attenuated CCI-mediated upregulation of TNF-α, IL-1β, and IL-6 expression and increased CCI-induced IL-10 mRNA levels, validating that depletion of CRNDE alleviated inflammation caused by CCI in the rats (Figure 3b). In summary, our data support the notion that CRNDE exacerbates neuroinflammation in CCI-induced NP.

## miR-146a-5p functions as a target of CRNDE

**Figure 4.**
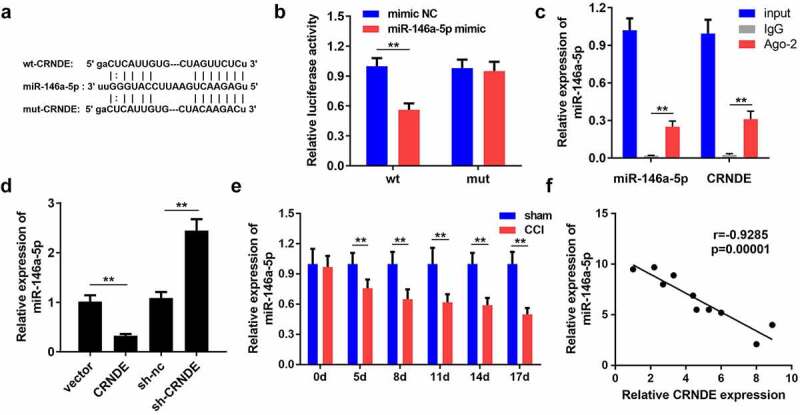
**miR-146a-5p functioned as a target of CRNDE**. (a) The potential binding sequences between miR-146a-5p and CRNDE predicted by starBase were shown. (b-c) Luciferase reporter assay and RIP experiment were used to assess the interaction between miR-146a-5p and CRNDE. (d) Transfect the CRNDE vector or sh-CRNDE into rat microglia, and use RT-qPCR analysis to estimate the role of CRNDE in miR-146a-5p expression. (e) The expression pattern of miR-146a-5p in rats following CCI surgery was determined by RT-qPCR. (f) Pearson correlation analysis of the relationship between miR-146a-5p and CRNDE. **P < 0.01

Therefore, we sought to understand the regulatory mechanism of CRNDE in the development of NP. Using the starBase database, we found that CRNDE can potentially to bind to miR-146a-5p (Figure 4a). Results from the luciferase reporter analysis showed that only the luciferase activity of CRNDE-WT was clearly decreased in response to a miR-146a-5p mimic, and no other significant changes were observed (Figure 4b). Similarly, high levels of miR-146a-5p and CRNDE were detected in the complexes immunoprecipitated with the Ago-2 antibody, which further confirmed the interaction between CRNDE and miR-146a-5p (Figure 4c). To determine the effect of CRNDE on miR-146a-5p, rat microglial cells were transfected with a CRNDE-expression vector or sh-CRNDE. RT-qPCR assay showed that overexpression of CRNDE significantly reduced the level of miR-146a-5p, while silencing of CRNDE enhanced miR-146a-5p expression (Figure 4d). In addition, the level of miR-146a-5p was lower in CCI rats with an increase in postoperative time (Figure 4e). Pearson correlation analysis revealed a negative association between CRNDE and miR-146a-5p (figure 4f). In summary, these experimental data demonstrated that miR-146a-5p was sponged by CRNDE.

## miR-146a-5p is negatively modulated by WNT5A

**Figure 5.**
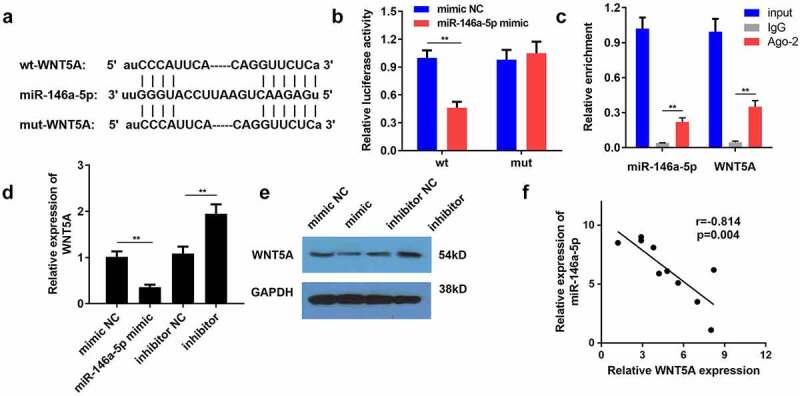
**WNT5A was negatively modulated by miR-146a-5p**. (a) By employment of bioinformatics tool starbase, WNT5A harbored speculated miR-146a-5p binding sites. (b-c) The interplay of miR-146a-5p with WNT5A was validated with luciferase reporter and RIP analyses. (d-e) The mRNA and protein expression levels of WNT5A were examined by RT-qPCR and western blotting in microglial cells. (f) The association between miR-146a-5p and WNT5A was analyzed by Pearson correlation analysis. **P < 0.01

Using the bioinformatics tool starBase, WNT5A was identified as a candidate protein downstream of miR-146a-5p. As shown in Figure 5a, WNT5A possessed the predicted binding sites of miR-146a-5p. We observed that co-transfection of WNT5A-WT and miR-146a-5p mimics markedly impaired the luciferase activity of HEK‐293 T cells (Figure 5b). The results of the RIP assay showed that miR-146a-5p and WNT5A were enriched in the IP with Ago-2 antibody compared to IP with IgG antibody, providing strong evidence that WNT5A directly bound to miR-146a-5p (Figure 5c). In addition, enhanced expression of miR-146a-5p resulted in a decrease in WNT5A mRNA levels in microglial cells, while repression of miR-146a-5p evoked the opposite effect (Figure 5d). Western blotting also demonstrated the inhibitory effects of miR-146a-5p on WNT5A expression at the protein level (Figure 5e). In agreement with the described findings, miR-146a-5p expression was negatively correlated with WNT5A expression (figure 5f). Thus, WNT5A serves as a downstream effector of miR-146a-5p.

## WNT5A knockdown retarded the NP progression *in vivo*

**Figure 6.**
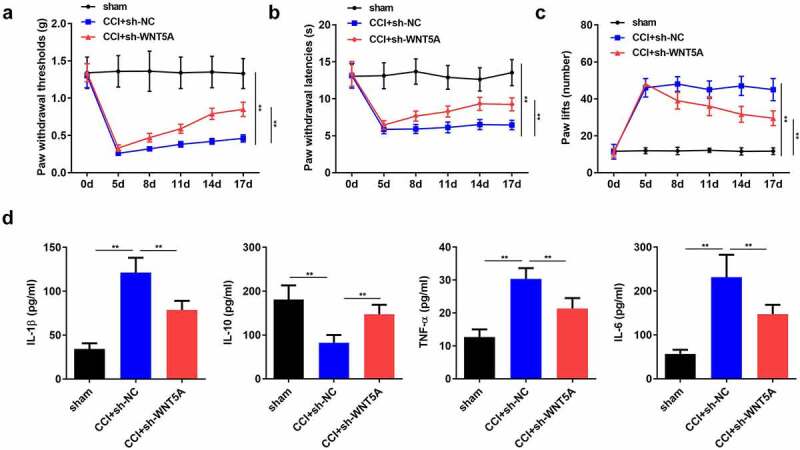
**WNT5A knockdown retarded the NP progression in vivo**. (a-c) Pain-related behavioral assessment was employed to evaluate the function of WNT5A silencing on the nociception of rats following CCI surgery. (d) ELISA for TNF-α, IL-1β, IL-6 and IL-10 expression in spinal cord tissues of rats from different groups. **P < 0.01

Next, we probed the function of WNT5A in nociceptive behaviors and inflammation in CCI rats. The results revealed that silencing of WNT5A alleviated mechanical allodynia and thermal hyperalgesia in rats subjected to CCI surgery (Figure 6a-c). Coincidently, ELISA suggested that the CCI-mediated marked increase in the concentrationsof IL-1β, TNF-α, and IL-6 was counteracted by downregulation of WNT5A (Figure 6d). Furthermore, CCI-mediated decrease in IL-10 level was restored by WNT5A suppression (Figure 6d). Overall, we confirmed that WNT5A promoted the development of NPs in CCI-induced rats.

## CRNDE accelerated the development of NP through targeting miR-146a-5p-mediated WNT5A in CCI rats

**Figure 7.**
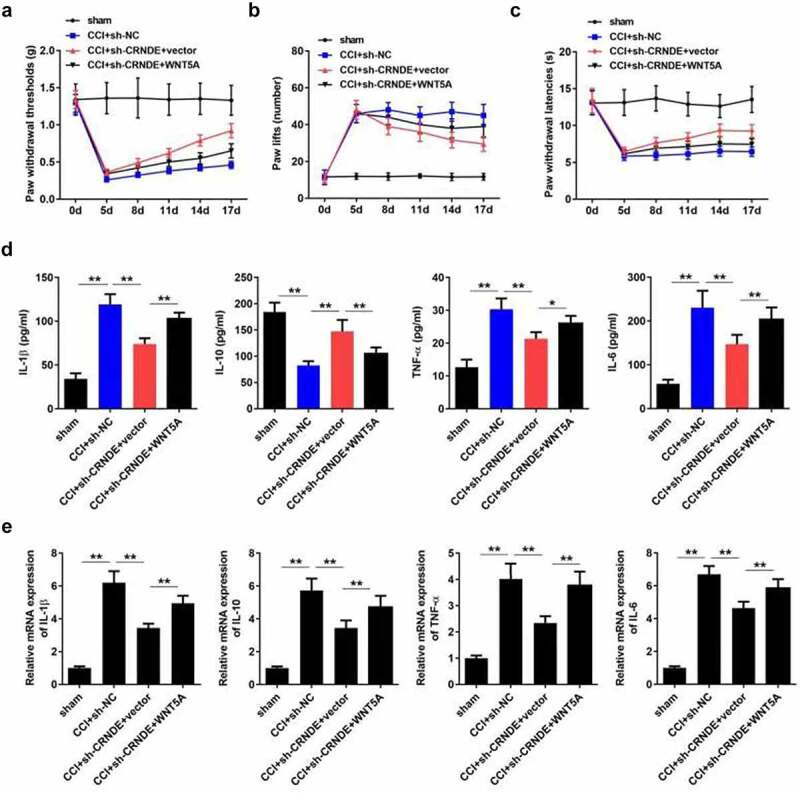
**CRNDE accelerated the development of NP through targeting miR-146a-5p-mediated WNT5A in CCI rats**. (a-c) The role of WNT5A in CRNDE-regulated mechanical allodynia and thermal hypersensitivity of CCI rats was estimated by pain-related behavioral assessment. (d) The neuroinflammation of CCI rats was evaluated by detecting TNF-α, IL-1β, IL-6, and IL-10 levels using corresponding ELISA kits. *P < 0.05, **P < 0.01

Lastly, we sought to confirm whether the function of CRNDE was mediated by the miR-146a-5p/WNT5A pathway. As expected, ectopic expression of WNT5A restored the threshold of mechanical allodynia, which was increased following depletion of CRNDE (Figure 7a). Consistently, overexpression of WNT5A abrogated the effects of CRNDE knockdown on thermal hyperalgesia in CCI rats (Figure 7b-c). Furthermore, the results showed that upregulation of WNT5A abrogated the CRNDE silencing-mediated inhibition of IL-1β, TNF-α, and IL-6 expression and restored IL-10 levels enhanced by CRNDE depletion (Figure 7d). Based on these results, we validated the role of CRNDE as a regulator of pain-related behavior and inflammation in CCI rats via the miR-146a-5p/WNT5A axis.

## Discussion

Neuropathic pain (NP) is one of the most intractable nervous system diseases, and its prevalence is growing prevalence [6,22]. Despite extensive efforts to understand the complicated etiology of NP, the efficacy of interventions currently available for NP remain inadequate [23,24]. Hence, it is important to better understand the molecular mechanisms underlying NP. To simulate NP *in vivo*, animal experiments commonly use the method of direct nerve damage, especially to the sciatic nerve [2,25–27]. Considering the involvement of lncRNAs in the pathophysiological process of NP, a rat model of NP induced by CCI was adopted in the current study to comprehensively clarify the characteristics and regulatory mechanisms of lncRNAs in NP development.

In the past few years, lncRNAs have become a research hotspot in the diagnosis and prevention of disorders of the nervous system [28,29]. Numerous studies have confirmed that lncRNAs play a crucial role in the etiopathogenesis of NP [30–32]. CRNDE has been extensively investigated in several malignant tumors and has been identified as a tumor promoting factor [33–36]. Furthermore, many studies have confirmed that CRNDE is involved in the regulation of neurobehavioral functions [37–39]. Importantly, recent research provides convincing evidence that CRNDE contributes to the exacerbation of CCI-induced NP in rats [16]. However, the detailed molecular mechanism of CRNDE in NP progression remains to be elucidated. In contrast to the sham control group, the expression level of CRNDE was markedly elevated in CCI-induced NP rats. Additionally, knockdown of CRNDE alleviated pain-related behavior and inflammatory response in CCI rats.

An increasing number of reports have illustrated that dysregulation of miRNAs is closely associated with the pathogenesis of diverse diseases [40–42]. Numerous studies have shown that miRNAs are of immense significance in neurophysiological and neuropathological processes [43,44]. Abnormal expression of miR-146a-5p has been observed in several disorders, such as human cancer, cardiovascular disease, and neuropathy [45–47]. Additionally, previous studies have validated that miR-146a-5p mitigates NP symptoms [48]. Considering that CRNDE harbors potential binding sites for miR-146a-5p, we selected it for subsequent investigations. As expected, our findings suggest that CRNDE serves as a sponge for miR-146a-5p. Moreover, WNT5A, which was reported to facilitate the development of NP [20,49], was found to be a downstream target of miR-146a-5p. Consistent with previous reports, knockdown of WNT5A alleviated the development of NP *in vivo*. Lastly, we verified that overexpression of WNT5A abrogated the effects of CRNDE-silencing on CCI-induced NP.

Collectively, to the best of our knowledge, the present study is the first to investigate the association between CRNDE, miR-146a-5p, and WNT5A in NP progression. Our experimental findings demonstrated that CRNDE upregulated WNT5A to attenuate pain-related behavior and inflammation in a CCI-induced rat model of NP, which suggests that CRNDE may represent an effective target for the diagnosis and treatment of NP.

## Conclusion

Our study found that the expression of lncRNA CRNDE was up-regulated in rats induced by chronic constrictive injury (CCI), and confirmed that miR-146a-5p is the target of CRNDE. In CCI rats, the expression of miR-146a-5p decreases, and at the same time it has a negative regulatory effect on WNT5A. We demonstrated that CRNDE up-regulates WNT5A through competitive binding with miR-146a-5p, thereby promoting the progression of CCI-induced NP.

## Highlights

1. Silencing CRNDE alleviates the development of NP in vivo.

2. CRNDE serves as a sponge for miR-146a-5p.

3. WNT5A is a target of miR-146a-5p.

4. CRNDE acts as a regulator in NP progression via the miR-146a-5p/WNT5A axis.
